# Supplementary material for: Nutrient Composition and Feed Hygiene of Alfalfa, Comparison of Feed Intake and Selected Metabolic Parameters in Horses Fed Alfalfa Haylage, Alfalfa Hay or Meadow Hay
Source: Animals (Basel). 2024 Mar 13;14(6):889. doi: 10.3390/ani14060889 (PMC10967314; doi:10.3390/ani14060889)
Supplement: Supplementary file 1 [file animals-14-00889-s001.zip › animals-2877969-supplementary.pdf]

Supplementary files

Table S1. Analytical ingredients of the mineral supplement as labelled.

**Analytical Ingredients**	**Percentage (%)**
Crude ash	47
Calcium	10
Phosphorus	2,5
Sodium	3
Magnesium	2.5
Starch	20
Sugar	5

Table S2. Urine parameters at different time periods for the feeding groups alfalfa hay (AH), alfalfa haylage (AS) and meadow hay (MH), data expressed as single values.

Parameter (in mmol/L)	Time	AH (*n*=6)	AS (*n*=5)	MH (*n*=4)
Calcium	Day 0–10	50.3/60.4	14.8/50.7/63.6	131.6
	Day 11–21	21.6/69.9/77.8/99.5	76.3/80.7	29.7/37.3/127.3
Phosphorus	Day 0–10	<LOD[1]/0.06	0.04/0.08/0.24	0.04
	Day 11–21	<LOD[1]/0.02/0.02/0.04	<LOD[1]	0.02/0.04/0.06
Chlorine	Day 0–10	141/143	99.2/113/148	116.6
	Day 11–21	79.6/80.3/90.1/156	66.4/120	97.7/118/169
Potassium	Day 0–10	425.5/484.5	168/455/470	390
	Day 11–21	406/423/444/500	323/377	331/380/452
Sodium	Day 0–10	14/14	16/19/69	13
	Day 11–21	13/15/15/15	11/14	11/13/14
Creatinine	Day 0–10	9.45/16.7	6.25/9.40/11.2	8.75
	Day 11–21	8.87/10.4/11.3/18.8	9.49/15.1	11.1/11.4/12.7

[1]LOD = Limit of detection.
